# Supplementary material for: Nested Biofabrication: Matryoshka-Inspired Intra-Embedded Bioprinting
Source: Small Methods. Author manuscript; Available in PMC 2024 Aug 18. (PMC11187694; doi:10.1002/smtd.202301325)
Supplement: Supporting Information [file NIHMS1978329-supplement-Supporting_Information.pdf]

small
methods

Supporting Information

for *Small Methods*, DOI 10.1002/smtd.202301325

Nested Biofabrication: Matryoshka-Inspired Intra-Embedded Bioprinting

*Mecit Altan Alioglu, Yasar Ozer Yilmaz, Yogendra Pratap Singh, Momoka Nagamine, Nazmiye Celik, Myoung Hwan Kim, Vaibhav Pal, Deepak Gupta and Ibrahim T. Ozbolat**

Supporting Information

Nested biofabrication: Matryoshka-inspired Intra-embedded Bioprinting

Mecit Altan Alioglu, Yasar Ozer Yilmaz, Yogendra Pratap Singh, Momoka Nagamine, Nazmiye Celik, Myoung Hwan Kim, Vaibhav Pal, Deepak Gupta, Ibrahim T. Ozbolat

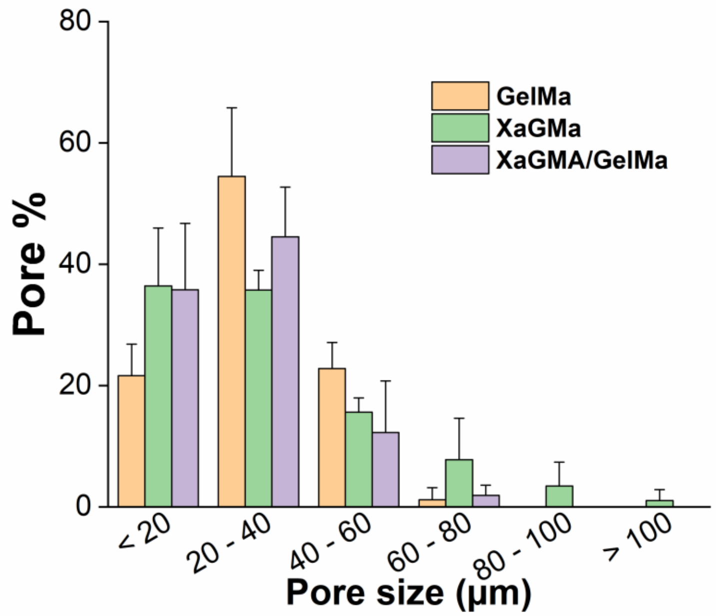

Figure S1. The pore size distribution in XaGMA, GelMA and the XaGMA/GelMA composite (*n*=3).

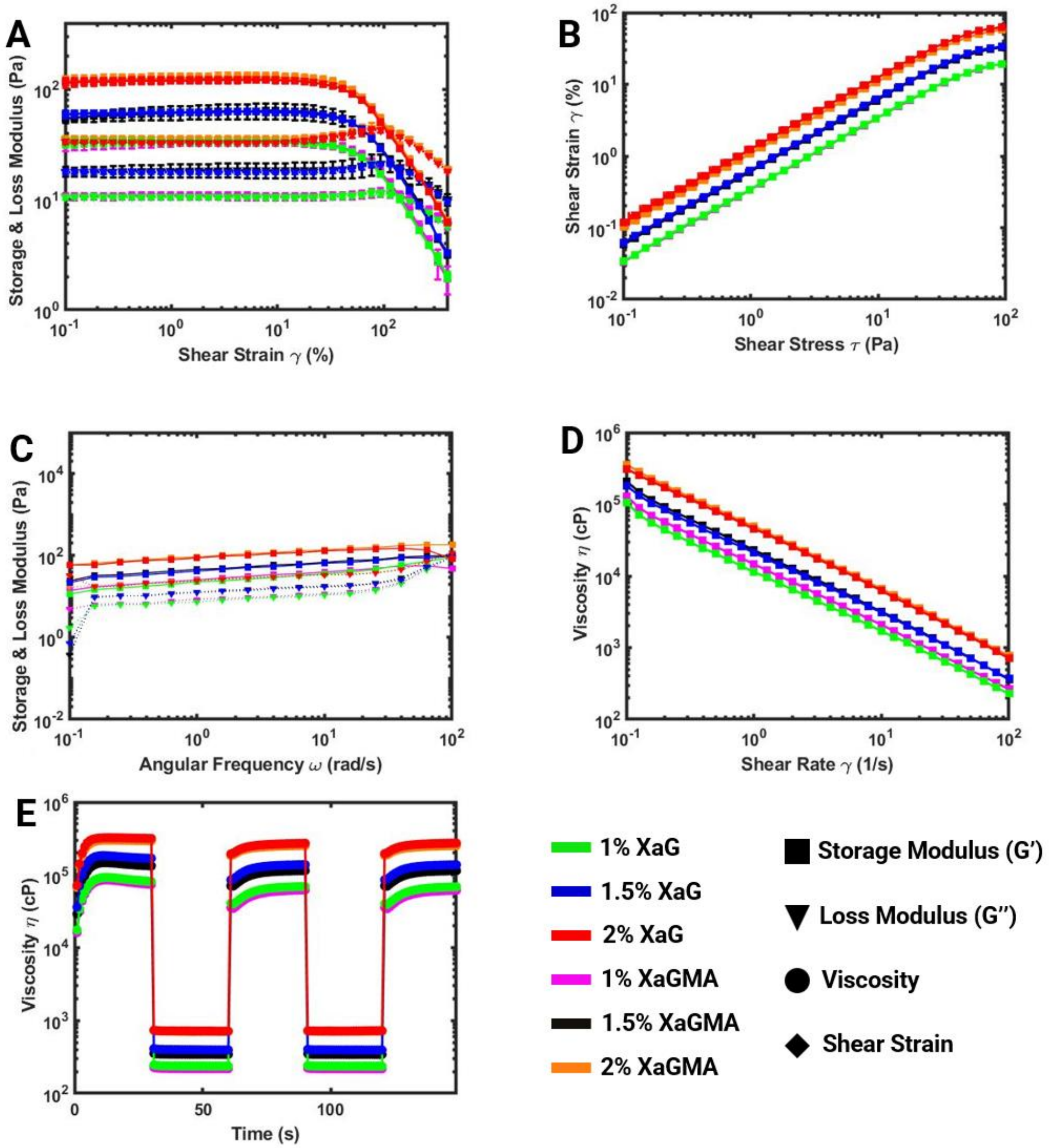

Figure S2. Rheological analysis of XaGMA. Rheology of XaG compared with the XaGMA composite, (A) amplitude sweep test, (B) stress vs. strain curve, (C) frequency sweep test, (D) flow curve, and (E) thixotropy test (n = 3).

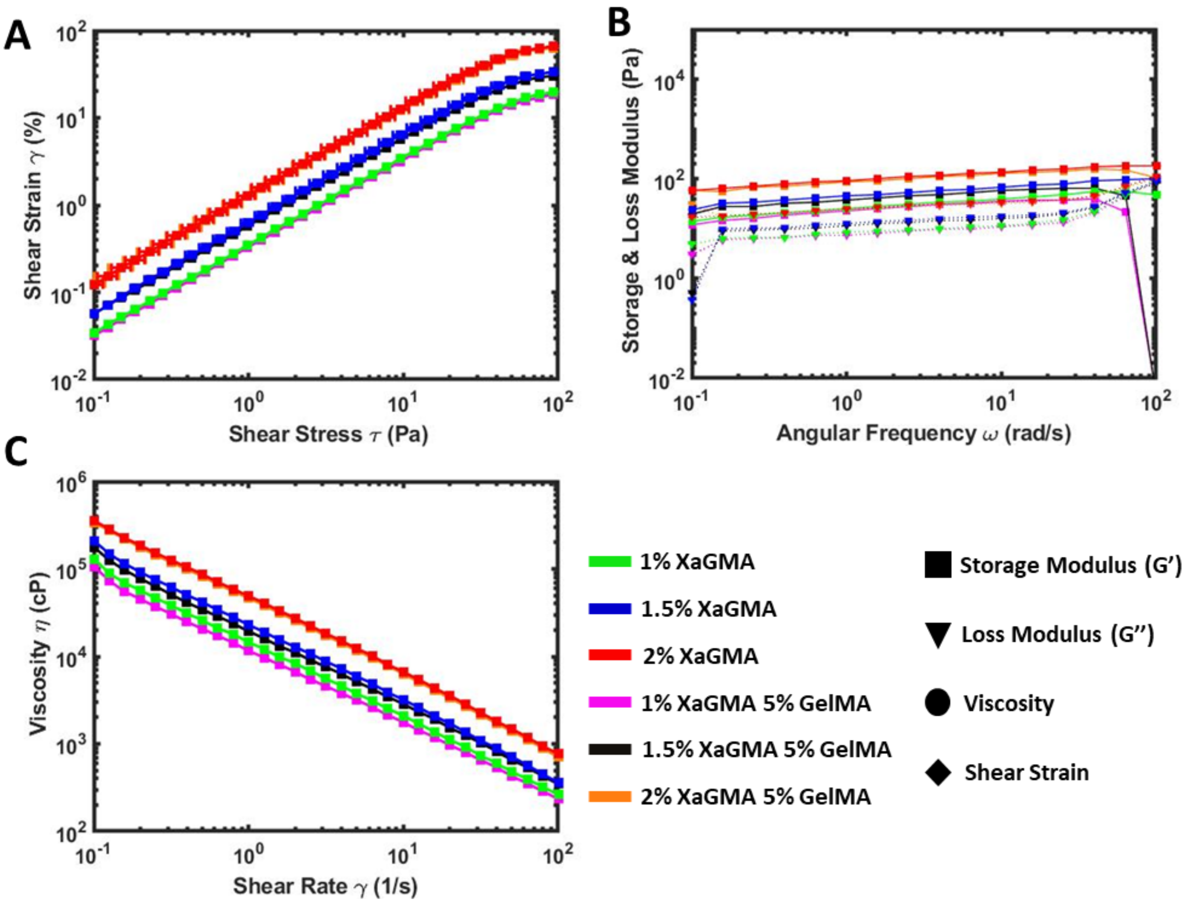

Figure S3. Rheological analysis of XaGMA/GelMA. (A) Stress-strain curve, (B) frequency sweep test to validate the elastic nature of the material at low shear strain and an angular frequency ranging from 0.1 to 100 rad s^{-1}, and (C) flow sweep test to measure the viscosity of the material under increasing shear rate (*n*=3).

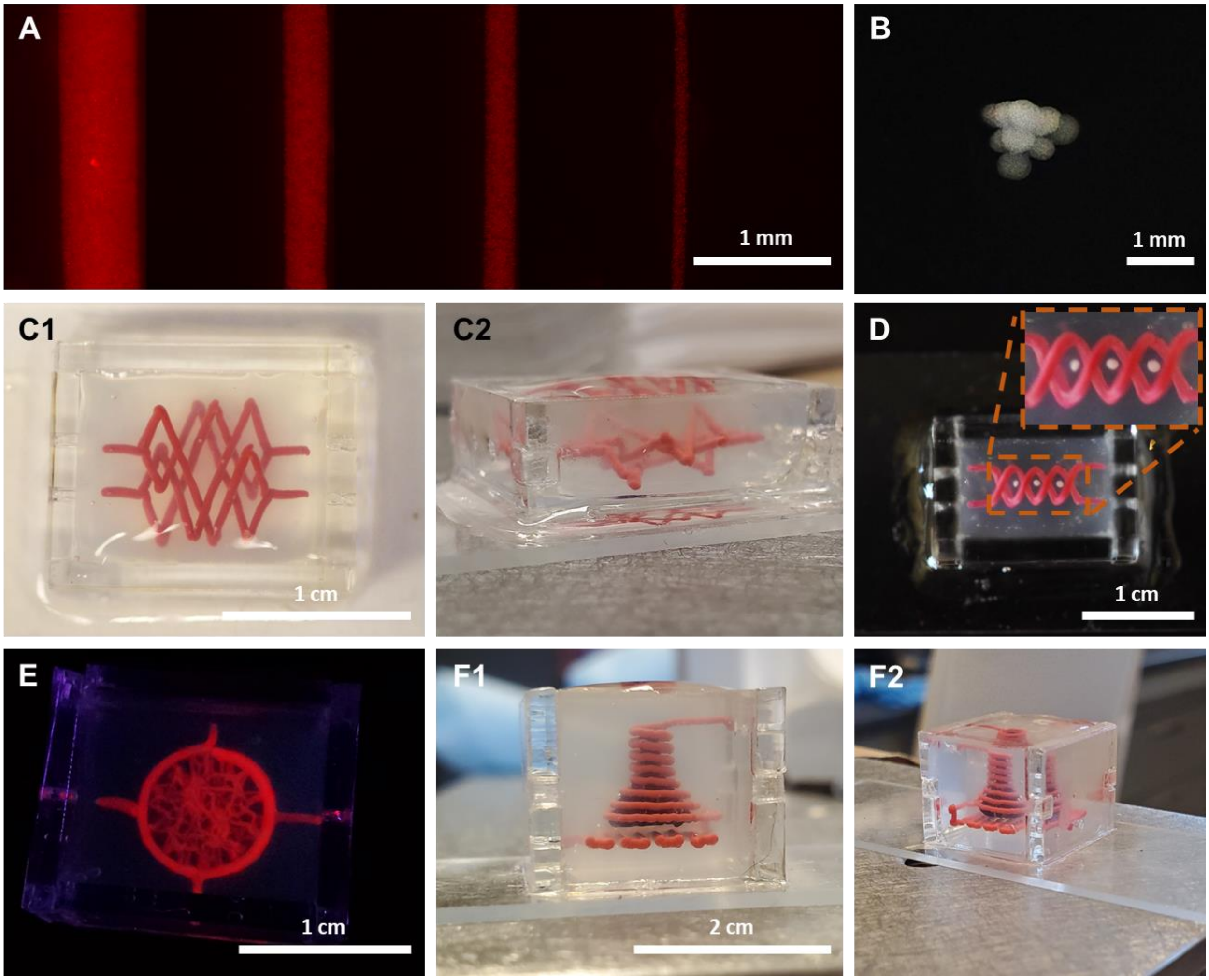

Figure S4. Embedded printing in XaG. Embedded printing of (A) filaments ranging from 100 to 600 µm, and (B) multiple spheroids in the shape of a pyramid. Embedded printing of (C1-C2) an intertwined channels design, (D) double helix channels with spheroids and (E) a bi-layer vascular bed design with a circumfential larger channel and smaller vascular channels within it. (F1-F2) Embedded printing of a lung model, where the alvelolar chamber was enclosed by a vasculature.

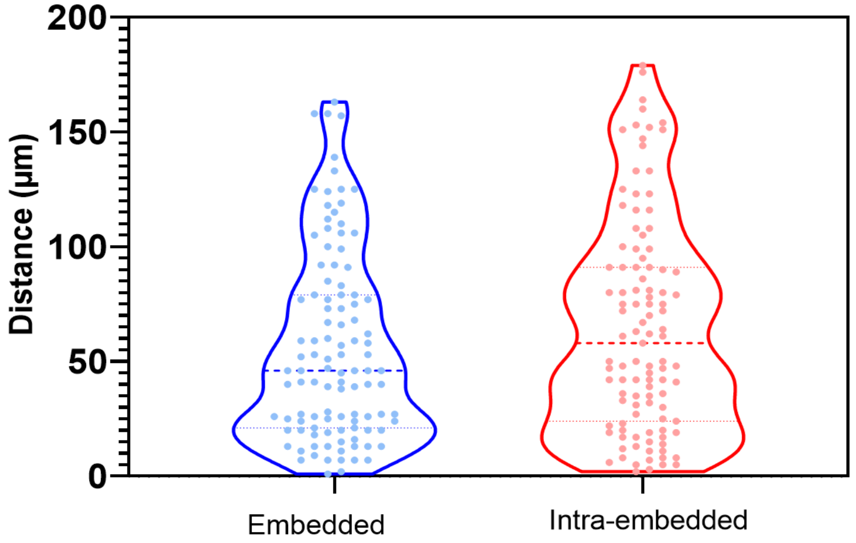

Figure S5. Accuracy and precision of embedded and intra-embedded (2nd nested structure inside the first one) printing. The positional accuracy and precision for dots bioprinted at 40 defined locations and a measured drift calculated between the designed and measured distance (*n*=3).

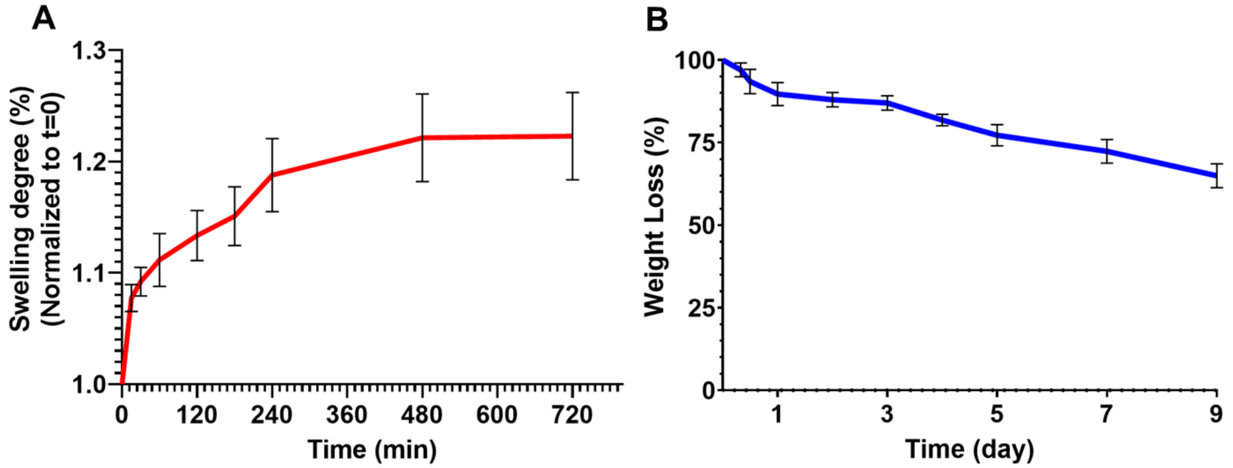

Figure S6. The stability of the printed constructs. (A) Swelling property (t=0 denotes time zero) and (B) degradation profile of the XaGMA/GelMA composite (*n*=4).

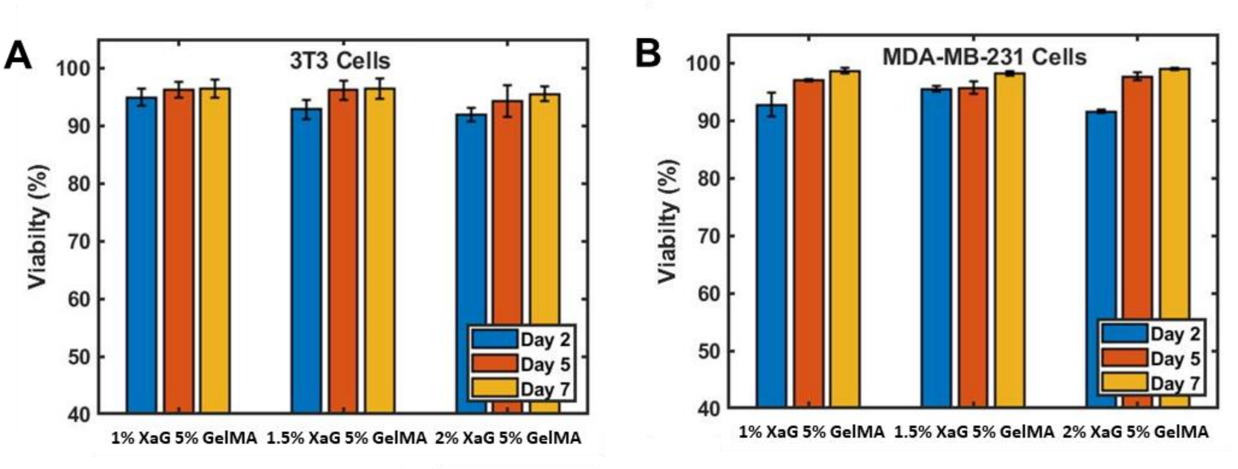

Figure S7. Biocompatibilty of XaG/GelMA. (A) Viability of 3T3 and (B) MDA-MB-231 cells on XaG/GelMA composites with different XaG concentrations (n=3).

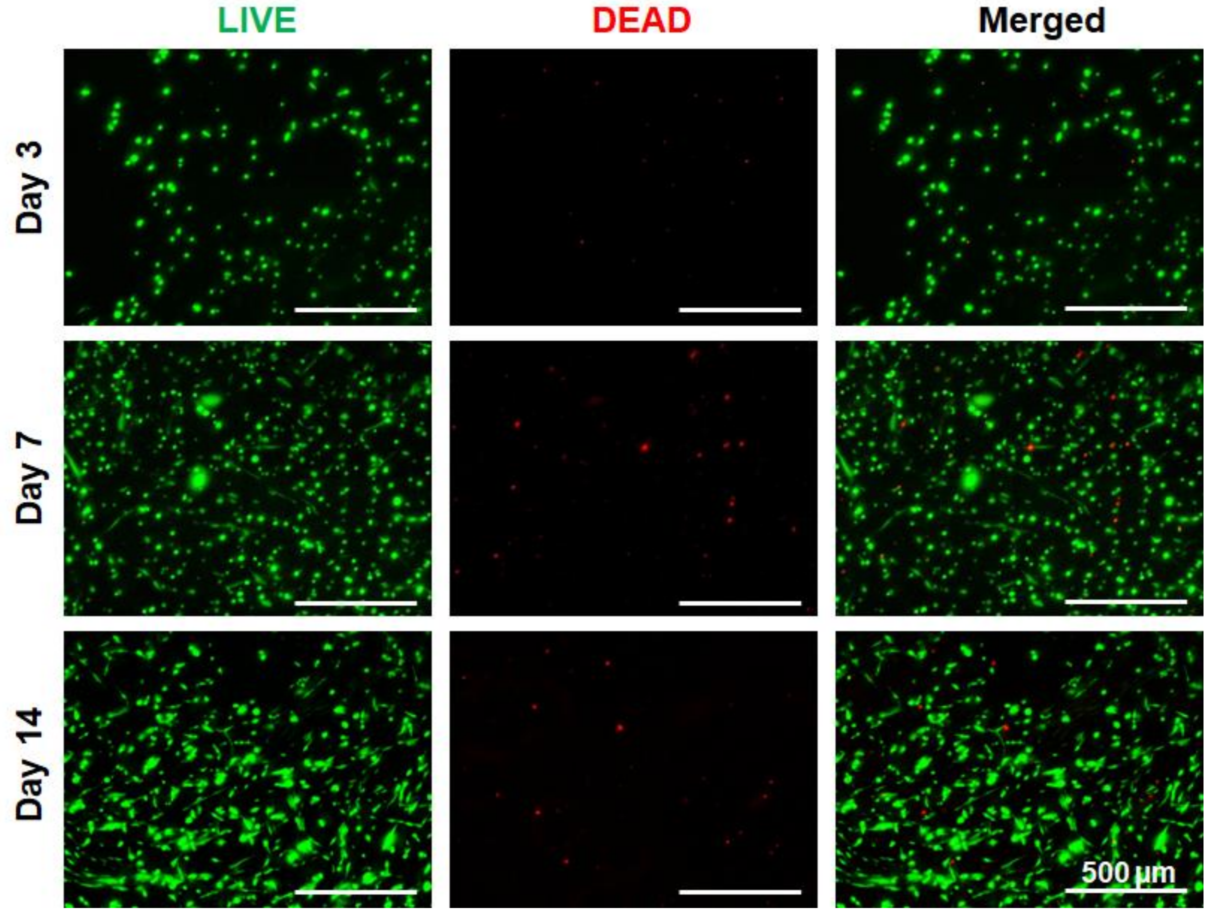

Figure S8. Viability of ADSCs in XaGMA/GelMA. LIVE/DEAD images of ADSCs on cast samples at Days 3, 7, and 14.

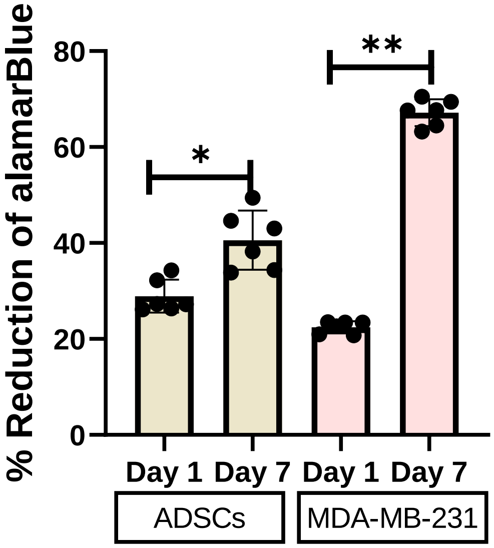

Figure S9. Cell proliferation assay. AlamarBlue assay showing the proliferation of ADSCs and MDAMB-231 cells on Days 1 and 7. Data were presented as mean ± S.D (n = 6; $^{*}p \leq 0.05$, $^{**}p \leq 0.01$).

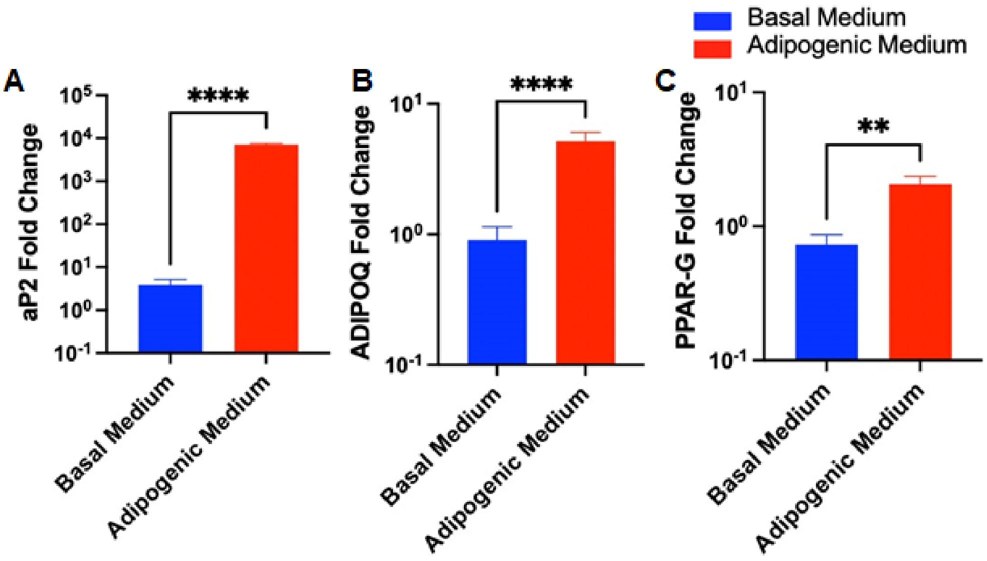

Figure S10. Gene expression of adipogenic markers using qRT-PCR on Day 15. (A) Adipocyte fatty acid-binding protein (aP2), (B) adiponectin (ADIPOQ), and Peroxisome proliferator-activated receptor gamma (PPAR-G). Data were presented as mean ± S.D. (n = 3; $^{**}p \leq 0.01$, $^{****}p \leq 0.001$).

Table S1. Primers of the genes used in qRT-PCR study.

Gene	Forward primer	Reverse primer
**aP2**	5'-ATG GGA TGG AAA ATC AAC CA-3'	5'-GTG GAA GTG ACG CCT TTC AT-3'
**PPAR-G**	5'-TCA GGT TTG GGC GGA TGC-3'	5'-TCA GCG GGA AGG ACT TTA TGT ATG-3'
**ADIPOQ**	5′-TGA CGA CAC CAA AAG GGC-3′	5′-GTG TGT CGA CTG TTC CAT GA-3′
**B-ACTIN**	5′-GCC CAC ATC TCC ACC TAT GAT-3′	5′-GCA GTT CTC GTT GTC CGT CA-3′

Movie Captions:

Movie 1: IEB of a pancreas model (10X playback speed)

Movie 2: IEB of a head phantom model (50X playback speed)

Movie 3: IEB of a Matryoshka doll (40X playback speed)

Movie 4: IEB of a cancer-on-a-chip model (10X playback speed)
